# Multidisciplinary management to optimize outcome of ultrasound-guided high-intensity focused ultrasound (HIFU) in patients with uterine fibroids

**DOI:** 10.1038/s41598-021-02217-y

**Published:** 2021-11-23

**Authors:** Florian Recker, Marcus Thudium, Holger Strunk, Tolga Tonguc, Sara Dohmen, Guido Luechters, Birgit Bette, Simone Welz, Babak Salam, Kai Wilhelm, Eva K. Egger, Ullrich Wüllner, Ulrike Attenberger, Alexander Mustea, Rupert Conrad, Milka Marinova

**Affiliations:** 1 Department of Gynaecology and Gynaecological Oncology, University Hospital Bonn, Bonn, Germany; 2 Department of Anaesthesiology, University Hospital Bonn, Bonn, Germany; 3 Department of Radiology, Städtisches Klinikum Solingen, Solingen, Germany; 4 Department of Diagnostic and Interventional Radiology, University Hospital Bonn, Bonn, Germany; 5 Center for Development Research (ZEF), University Bonn, Bonn, Germany; 6 Department of Radiology, Johanniter Krankenhaus Bonn, Bonn, Germany; 7 Department of Neurology, University Hospital Bonn, Bonn, Germany; 8 Clinic and Polyclinic for Psychosomatic Medicine and Psychotherapy, University Hospital Bonn, Bonn, Germany; 9 Clinic for Diagnostic and Interventional Radiology, University Hospital Bonn, Venusberg-Campus 1, 53127 Bonn, Germany

**Keywords:** Outcomes research, Signs and symptoms, Reproductive signs and symptoms

## Abstract

Little is known about the specific anaesthesiological and multidisciplinary management of high-intensity focused ultrasound (HIFU) in uterine fibroids. This observational single-center study is the first reporting on an interdisciplinary approach to optimize outcome following ultrasound (US)-guided HIFU in German-speaking countries. A sample of forty patients with symptomatic uterine fibroids was treated by HIFU. Relevant treatment parameters such as total treatment time for intervention, anaesthesia, and sonication time as well as total energy, body temperature, peri-interventional medication and complications were analyzed. Interventional variables did not correlate significantly either with opioid dose or with body temperature. The average fibroid volume reduction rate was 37.8% ± 23.5%, 48.5% ± 22.0% and 70.2% ± 25.5% after 3, 6 and 12 months, respectively. No major anaesthesiological complications occurred apart from an epileptic seizure prior to HIFU treatment in one patient. Peri-procedural hyperthermia (> 37.5 °C) occurred in two patients. Post-procedural two patients experienced a sciatic nerve irritation up to one year; one patient with very large treated fibroid experienced strong short-lasting post-procedural pain. There were two complication-free pregnancies of HIFU-treated patients. Multidisciplinary management is crucial to optimize safety and outcome of US-guided HIFU for uterine fibroids. Peri-procedural pain and temperature management are critical points where an adequate collaboration between anesthesiologist and interventionalist is mandatory.

## Introduction

Uterine fibroids are the most common benign tumors among women in the reproductive age. The main surgical therapy options involve laparoscopic or open myomectomy, and hysterectomy. Other approaches include medical treatment with mifepristone, radiofrequency ablation of the uterine fibroid or uterine artery embolization (UAE)^1^. In the last decades, several attempts have been made to develop alternative therapy options. In the recent years, high-intensity focused ultrasound (HIFU), either guided by ultrasound (USgHIFU) or by magnetic-resonance tomography (MRgHIFU), seems to be an effective option for uterine fibroid management^2,3^. HIFU is a non-invasive technology that can be effectively used in a wide range of clinical applications for the treatment of neurological, genitourinary, hepato-biliary, musculoskeletal, and oncological diseases ^4^. Using HIFU uterine fibroids are thermally ablated via non-invasive approach by concentrating ultrasound energy on a small tissue area, causing coagulation necrosis and destruction of selected tissue^5,6^. In contrast to other local ablative methods, the main advantage of HIFU lies in its noninvasiveness: the treatment does not involve use of needles, probes, or electrodes. With a very low complication rate, HIFU provides an effective treatment option in patients suffering from fibroid-associated symptoms. As the procedure itself has some special features, it requires a precise coordination between interventionalist, e.g., interventional radiologist, gynecologist, and anesthesiologist during ablation to enhance accuracy of treatment and ensure patient safety. In Germany a consensus conference stated that the indication for treatment of uterine fibroids should be determined in an interdisciplinary manner following by a gynecological examination and counseling of the patient. Comprehensive patient counseling regarding different treatment options for symptomatic uterine fibroids encompasses not only medication-based and surgical, but also non-surgical treatment options^7,8^. Based on data of HIFU-treated patients with uterine fibroids in our hospital, this study aimed to evaluate relevant factors in the multidisciplinary management in order to facilitate treatment, anticipate and avoid potential physiological derangements or injury and coping with the intraprocedural pain. To the best of our knowledge such data on USgHIFU of symptomatic uterine fibroids are reported for the first time from German-speaking countries and Europe apart from a small study from Oxford with 12 HIFU-treated patients in 2019^9^.

## Methods

### Patient cohort

**Figure 1:**
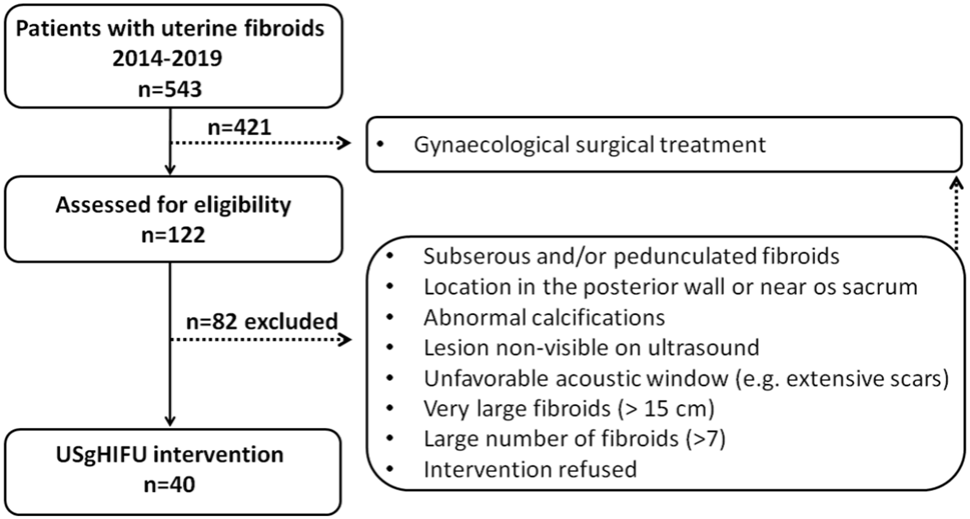
Consort diagram: assessment for eligibility and USgHIFU intervention.

**Figure 2:**
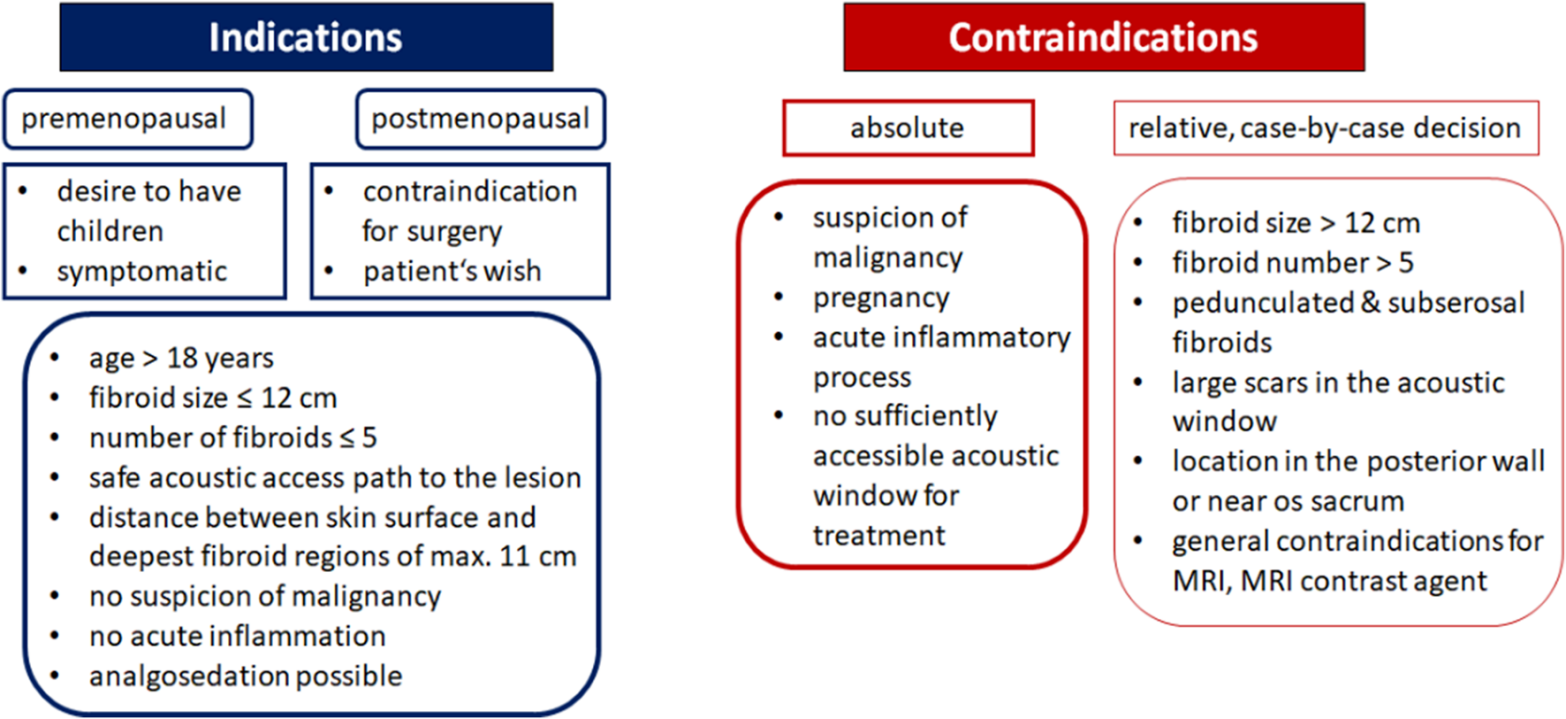
Indication and contraindication for HIFU treatment of uterine fibroids.

Between 2014 and 2019, 543 patients with symptomatic uterine fibroids presented at the department of gynaecology and radiology at the university hospital Bonn and underwent a therapeutic procedure (Fig. 1). Among these women, 40 patients were treated with USgHIFU; the clinical indication was confirmed by an interdisciplinary board for each patient (Fig. 2). The study was performed in accordance with the Declaration of Helsinki and approved by the local ethics committee of the medical faculty of the Rheinische-Friedrich-Wilhelms-University of Bonn (No. 302/12; 307/15). A written informed consent was obtained from each patient.

### Pretreatment procedures and HIFU intervention

Pretreatment evaluation included medical history, physical examination and laboratory tests. Due to the lesion proximity to some parts of the gastrointestinal tract, a specific bowel preparation is required in some cases with relatively small fibroids to avoid possible complications. The bowel preparation starts one day before the procedure and consists of liquid food, no gas producing food and fasting for 12 h^10,11^. The skin of the patient´s lower abdominal wall is shaved, degreased and degassed. USgHIFU ablation was performed using the Focused Ultrasound Tumor Therapeutic System (JC HIFU, Chongqing HAIFU Technology, China) equipped with a 1–8 MHz ultrasound imaging device (MyLab 70, Esaote, Italy) for real-time guidance. The therapeutic ultrasound beam was transmitted by a 20 cm diameter ceramic transducer with a focal length of 15 cm, operated at a frequency of 0.8 MHz. The design of the HIFU system with the water basin and transducer below the table requires a treatment in prone position. Water is used as a coupling medium between the ultrasound device and patient’s skin as well as for cooling the skin of the lower abdominal wall. For planning and ablation a sagittal scanning mode is used; US energy is delivered to a circumscribed focal area using a dot mode. Repeated cycles of 1 s sonication followed by a 3 s break were delivered at each focal point. In case of visible grey-scale changes in the target area suggesting effective ablation the transducer is moved to the next focal zone in the same slide, then in adjacent slides in order to achieve volume ablation. A safety margin of 1 cm to existing structures at risk (bowel parts) is maintained to prevent potential damage by local temperature increase. The volume ablation is composed of multiple focal sonications in rows and adjacent layers. The applied power is adjusted individually for every patient. During the intervention the skin is kept in cooled water of 15–20 °C and examined regularly by palpation. The follow-up imaging includes contrast-enhanced MRI (1.5-T. Ingenia MRI, Philips Healthcare, the Netherlands) for all patients.

### Anaesthesia

USgHIFU treatment of uterine fibroids is performed in analgosedation. Preparation for anaesthesia includes preoperative evaluation and informed consent according to current standard procedures at least 24 hours prior to intervention. Since there is no standard sedation regimen, the choice of anaesthetic agents is left to the discretion of the individual anaesthesiologist. Once in the intervention room, patient received ASA standard monitoring and one peripheral cannula. Oxygen is applied and expiratory CO_2_ was measured via a nasal probe. Sedation is performed with the intention that the patient tolerates positioning and intervention but is at the same time conscious enough to report major pain events and/or irritation of nerval structures (e.g. sciatic nerve) in close proximity to the ultrasound focus.

### Statistical analysis

Statistical analysis was performed with Stata 16 Software (StataCorp. 2019. Stata Statistical Software: Release 16. College Station, TX: StataCorp LLC) using a mixed linear data model. Mean, median, standard deviation (SD), range and exact 95% confidence intervals (CI) were calculated. The primary statistical evaluation of fibroid volumes was performed using mixed model considering values at baseline and each follow-up as dependent variables^12^. For correlation between two continuous variables, the Spearman’s correlation test was performed. Results were considered statistically significant if the p-value was < 0.05.

## Results

### USgHIFU treatment

Fourty female patients (aged 28–53 years) with more than 50 uterine fibroids (largest diameter 2–12 cm) were successfully treated by USgHIFU at our institution. Patients’ characteristics are summarized in Table 1.

**Table 1:** Patients’ characteristics of 40 patients with symptomatic uterine fibroids (UF) treated with US-guided HIFU at our institution.

Parameter	Value
Patients, n	40
Age, mean ± SD (range)	42 ± 6.2 (28–53)
Body size (cm), mean ± SD (range)	167.7 ± 6.3 (154–179)
Body weight (kg), mean ± SD (range)	64.3 ± 9.3 (45–85)
BMI, mean ± SD (range)	22.8 ± 3.1 (18.3–29.4)
Fibroid volume (ml), mean ± SD (range)	88.15 ± 84.0 (4.5–332.3)

A single USgHIFU session was performed in the majority of patients (n = 38/40) (Table 2), five of these patients presented after unsuccessful (n = 3) or refused (n = 2) MRgHIFU at other institutions. Due to recurring symptoms, HIFU was repeated in two patients (at 16 and 7 months after 1st session). In the post-interventional course, one patient underwent uterine artery embolization (UAE) 40 months after HIFU procedure, two other patients a gynaecological surgery, abdominal hysterectomy and laparoscopic hysterectomy, each 17 months after USgHIFU. There was no need for peri-interventional discontinuation of any concurrent medication (e.g. anticoagulant drug in one patient) as it is the case in surgery. In our cohort two patient gave complication-free birth after USgHIFU treatment.

**Table 2:** Therapeutic parameters of US-guided HIFU treatment in patients with uterine fibroids (n = 40).

Parameter	Value
Treatment time (min)	167.6 ± 44.7 (69–275)
Sonication time (s)	1147 ± 445.7 (289–2013)
Total energy (kJ)	328.5 ± 148.8 (81.2–586.9)
Average power (W)	290 ± 73 (105–400)
Energy/per ml firboid volume	8.6 ± 10 (1.1–46.9)

### Anaesthesia during HIFU procedure

In a retrospective chart review, radiological and anaesthesiological records were screened for used anaesthesia, pain medication and dose, highest and lowest body temperature measured during HIFU procedure, intravenous fluids, minimal oxygen saturation, and intra- and postinterventional complications. Postinterventional opioids were calculated into morphine equivalents. Several regimes of analgosedation could be identified. All patients received continuous remifentanil between 0.48 and 5.55 µg/kg/min. The mean dose was 2.06 µg/kg/min. Eleven patients received remifentanil alone, 13 patients received a combination with dimenhydrinate (mean dose 62.6 mg), and another 16 patients received a combination with propofol (mean: 1.64 mg/kg/h). Propofol was the predominant regime in the beginning of the HIFU procedure and was additionally combined with S-Ketamine in two patients. Postoperative analgesia was performed with piritramide in 21 patients with a maximal dose of 21 mg (30 mg morphine equivalent; 0.58 mg/kg bodyweight morphine equivalent) (Table 3). A statistically significant correlation between lesion volume to be treated and intervention parameters such as treatment time (p < 0.001, rho = 0.71), sonication time (p = 0.001, rho = 0.70) and total energy (p < 0.001, rho = 0.70) was demonstrated. No significant correlation was found between volume reduction following USgHIFU and mentioned intervention parameters (treatment time: p = 0.20, r_s_ = 0.42, sonication time: p = 0.10, r_s_ = 0.52, total energy: p = 0.25, r_s_ = 0.38). Initial fibroid volumes (p = 0.53, r_s_ = 0.42) or volume reduction rate after one year (p = 0.08, r_s_ = 0.55) did also not correlate with postinterventional opioid consumption.

**Table 3:** Peri-procedural anaesthesia during US-guided HIFU procedure.

Drug	Value
**Remifentanil (µg/kg/min)**	
Number of patients	40 (100%)
Mean dosis ± SD (range)	2.06 ± 0.18 (0.48–5.55)
**Exclusive use of remifentanil**	
Number of patients	9 (22.5%)
**Additional dimenhydrinate (mg)**	
Number of patients	13 (32.5%)
Mean dosis	62.6
**Additional propofol (mg/kg/h)**	
Number of patients	16 (40%)
Mean dosis	1.6
**Postoperative piritramide**	
Number of patients	21 (52.5%)
Mean dosis (mg)	9.36
Min. dosis (morphine eq.)	3
Max. dosis (morphine eq.)	21

During intervention the lowest registered oxygen saturation was 90% (mean value: 96.14%, SD: 2.11). The lowest body temperature was 35.6 °C and the highest was 38.3 °C, measured in patient’s ear. The observed temperature differences (mean 0.84 °C ±0.6 °C) did not correlate significantly either with the total applied energy (p = 0.64), applied energy per ml volume (p = 0.91), sonication time (p = 0.76) or treatment time (p = 0.51). The patients' BMI (p = 0.23) and age (p = 0.23) also seem not to have an influence on temperature ranges. Around 50% of the patients shivered within next 30 min directly after the HIFU procedure resulting in an intravenous administration of clonidine (75–150 µg in one or two boluses).

### Fibroid volume after USgHIFU

**Figure 3:**
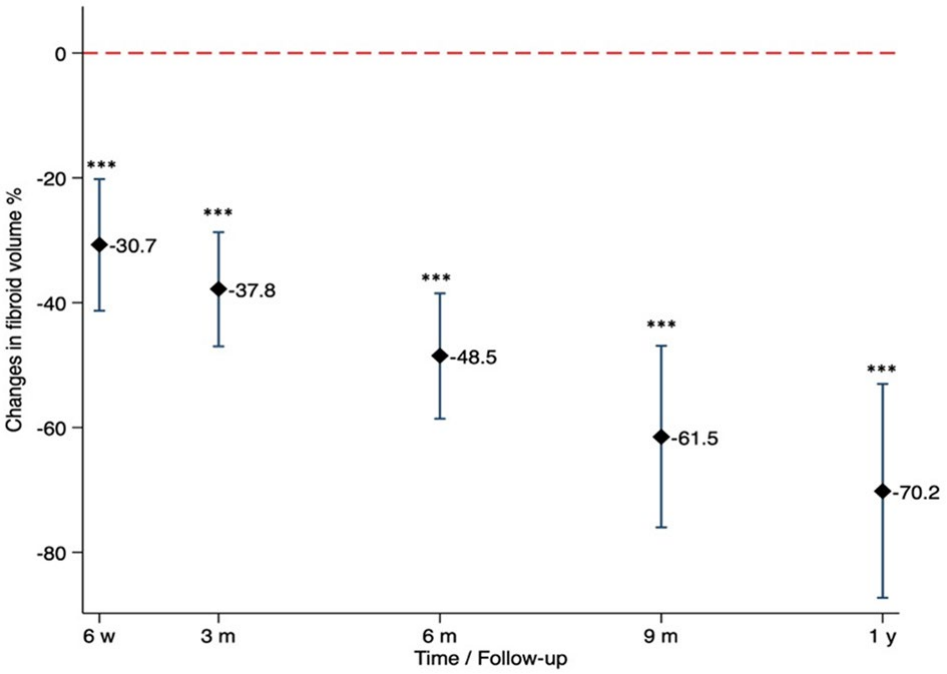
Changes in fibroid volumes during follow up. Significant fibroid volume reduction rate was observed 6 weeks after HIFU compared to baseline (95% CI: − 52%, − 74%, p < 0.05). Lesion shrinkage improved continuously over the observational period at 3-, 6-, 9-month and 1-year follow-up (each p < 0.001, compared to baseline, mixed model). The figure was created using Stata 16 Software (StataCorp. 2019. Stata Statistical Software: Release 16. College Station, TX: StataCorp LLC).

**Figure 4:**
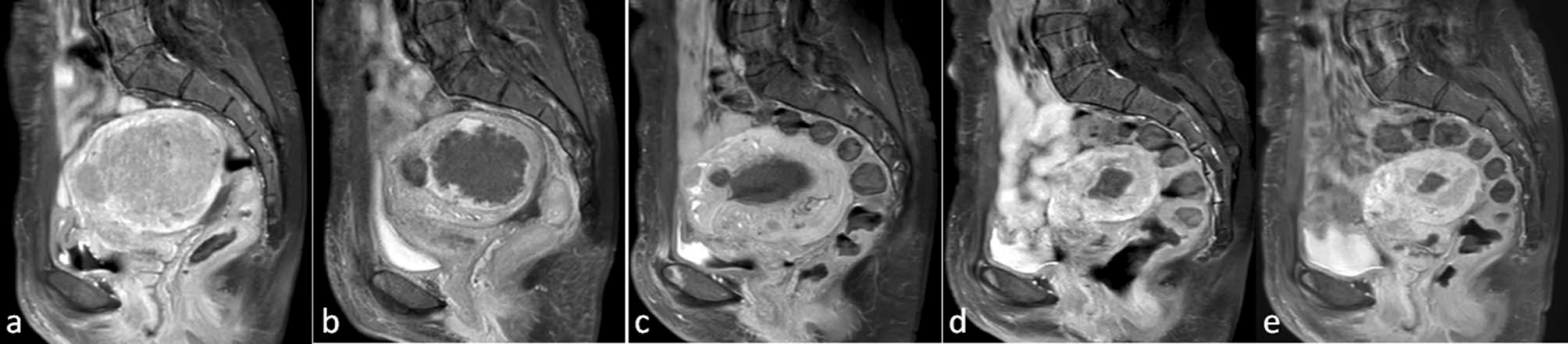
A 45-year old patient with symptomatic uterine fibroids presented with menstrual complaints (hyper-dysmenorrhea), urination urge, pain/pressure in the pelvic region and was treated by USgHIFU in our hospital. Three weeks after HIFU, considerable symptom relief was observed. Representative MRI-images (**a**–**e**, contrast-enhanced T1-weighted in sagittal plane) of the largest uterine fibroid are shown over time before and after HIFU treatment: (**a**) baseline with initial fibroid size of 6.2 × 5.4 cm; (**b**) one day post-HIFU large treated region showed no contrast enhancement indicative of effective ablation*;* (**c**–**e**) A continuous reduction in fibroid volume after HIFU-treatment was observed, this was 66.4%, 84.6% and 90.1% after 6 months, 1 and 2 years, respectively.

The fibroid volume was measured during follow up on T2-weighted MRI at 6 weeks, 3, 6, 9 and 12 months after HIFU treatment. An average non-perfused volume rate (NPVR) of 58.4% ± 31.8% was observed in first contrast-enhanced post-interventional imaging (T1-weighted MRI) directly after HIFU as described previously^11^. The reduction rate of lesion volume over time compared to initial values is shown in Table 4, Fig. 3, and representative MRI of HIFU-treated patient in Fig. 4. In total, the volume reduction rate of HIFU-treated fibroids averaged to 37.8% ± 23.5%, 48.5% ± 22.0% and 70.2% ± 25.5% after 3, 6 and 12 months, respectively (Table 4).

**Table 4:** Lesion volumes of HIFU-treated uterine fibroids at baseline and follow-up (6 weeks, 3, 6 and 9 months, 1 year after HIFU); corresponding volume reduction in % compared to initial lesion volumes.

	Fibroid volume (ml)	Volume reduction rate (%)
Baseline	88.1 ± 84.0^a^ (4.5; 332.3)	
6-week FU	58.6 ± 51.5 (2.1; 162.2)	30.7 ± 21.2
3-month FU	56.0 ± 51.5 (2.1; 162.2)	37.8 ± 23.5
6-month FU	55.35 ± 45.9 (5.4; 142.9)	48.5 ± 22.0
9-month FU	33.5 ± 44.2 (0; 146.6)	61.5 ± 24.0
1-year FU	24.8 ± 24.8 (2.9; 81.9)	70.2 ± 25.5

^a^Mean ± standard deviation (range).

### Side effects and complications

In one patient, an epileptic seizure occurred when sedation was initiated before the start of HIFU treatment. This was retrospectively associated with an allergic reaction to an unknown agent and rated as serious sedation-related adverse event and anaesthesiological complication. The patient was transferred to the intensive care unit for further treatment. No other major anaesthesiological complications occurred in our patient cohort. Regarding HIFU-related and gynecological adverse events, no major complications were observed in majority of patients (95%). Peri-interventional short-lasting side effects included vaginal bleeding (5%), transient short-lasting abdominal pain (15%), increased vaginal discharge (6%), and subcutaneous edema of the lower abdominal wall (30%). These side effects were self-limiting or treated symptomatically by over the counter medication (NSAI, paracetamol). Post-procedure, two patients experienced irritation of the sciatic nerve with radiation into the leg, in one patient this was self-limiting without specific medication, in the other patient it was lasting for up to one year requiring anti-inflammatory drugs. One patient with very large treated fibroid experienced strong post-procedural pain; this patient was transferred to the intermediate care unit of the department of gynaecology for one night receiving a Würzburg pain drip consisting of tramadol, metamizole and droperidol. No further adverse events were observed.

## Discussion

Many studies from Asia with more than 10,000 treated patients^2,3,13^ as well as one study from Europe with 12 patients^9^ have demonstrated in more recent times that USgHIFU is a safe and effective alternative for treatment of uterine fibroids. This study is the first reporting on multidisciplinary especially peri-procedural anaesthesiological management following USgHIFU of uterine fibroids in German-speaking countries.

Our study lends further evidence for the fact that in the HIFU setting, peri-procedural temperature management represents a challenge for both, the anesthesiologist and the interventionalist. In our patient cohort, eight patients experienced hypothermia and two patients hyperthermia. HIFU ablation causes local hyperthermia inside the tumor in order to destroy tissue with the focused energy of the ultrasound beam. On the other side, the abdominal wall has to be cooled during the procedure to prevent skin burn and injury, which is especially important in patients with scars. There was no significant association between applied energy and body temperature, indicating that in the majority of patients the body temperature could be kept constant regardless of the energy used. Therefore, a close monitoring of temperature is crucial no matter which energy is applied^14^. Our results underline the necessity of a close inter-disciplinary collaboration to avoid excessive hypo- or hyperthermia which could have a negative influence on patients’ well-being during the procedure. The results also suggest that temperature loss represents a more common issue than hyperthermia caused by the HIFU procedure. We attribute this to a combination of impaired thermoregulation under sedation and cold exposure, which is commonly observed in the operative setting^15^. As the patient's anterior lower abdominal wall is placed in a water bath, water spilling is possible and may lead to additional temperature loss through evaporation despite constant warming by a warming blanket. About half of our patients experienced body shivering directly post-intervention, which can be considered as a procedure-associated concern and was successfully managed by the anaesthesiologist by adequate intravenous administration of clonidine. Pre-warming may reduce the incidence of hypothermia as it has been shown in the surgical setting^16^.

Sedation for HIFU in prone position represents another challenge from anesthesiologist’s point of view. Deep sedation may lead to desaturation events or unnoticed injury by the HIFU therapy^17^. Therefore, patients have to be conscious enough to be able to report severe pain or sciatic nerve irritation but at the same time have to tolerate prone position and the treatment itself for a prolonged period of time (e.g. up to 4–5 h in cases with huge fibroids). In all patients the applied regime was based upon continuous remifentanil combined in about a third of patients (13, 32.5%) with a dimenhydrinate bolus, in 16 patients (40%) with propofol. This is in line with the literature in which no single method of sedation is superior^17^. However, it has to be noted that while providing good pain control, remifentanil carries an increased risk of respiratory depression^18^. Since the lowest recorded oxygen saturation was 90%, all applied regimes can be considered safe. However, temporary hypoventilation may still have occurred requiring adjustment of sedation and possibly breathing commands. While it was not routinely used in our patients, processed electroencephalography (pEEG) monitoring may be considered to guide and refine depth of sedation which has been shown to be effective and which may further increase safety^19^. Longer cases may benefit from pEEG feedback to avoid saturation effects, especially when propofol is used. However, it has been shown that pEEG accuracy is decreased in a remifentanil-based regime^20^. Therefore it remains to be evaluated whether pEEG is sufficient to monitor adequate analgesia with remifentanil or if separate analgesia monitoring is needed^21^.

Post-procedural pain can be another challenge although this is usually self-limiting and often requires only over the counter medication, e.g. NSAI, paracetamol, hyoscine-butylbromide. In our cohort, two patients showed a sciatic nerve irritation with onset on the first day after HIFU procedure lasting up to one year. Only one patient experienced strong post-procedural pain similar to the reported post-embolisation syndrome after UAE^22^. This patient, who was treated for a very large fibroid, stayed on the intermediate care unit of the department of gynaecology for one night and pain symptoms were successfully treated by pain drop consisting of tramadol, metamizole and droperidol.

Interestingly, no significant relationship was found between lesion volume to be treated, intervention parameters (such as sonication time and total energy) and post-interventional opioid consumption. Nonetheless, over 50% of patients required analgesic treatment by piritramide post-intervention. In some patients in the first hour post-intervention high doses of piritramide comparable to those for HIFU-treated patients with pancreatic cancer were necessary^23^, however this intensified analgesic regimen could not be predicted from fibroid or intervention characteristics. Against this backdrop large-sized studies monitoring even more clinical and interventional variables are urgently needed to hopefully identify specific subgroups of patients regarding peri- and post-interventional analgesic treatment. On the other hand, the innovative application of objective methods of pain detection may be very helpful to identify and treat pain symptoms at a very early peri- or postprocedural stage and, thus, avoid higher doses of opioids^21^. These could also be applied during general anesthesia, thus providing an objective measure of intraprocedural nociception without the need for a responsive patient, thus possibly avoiding the potential risks of sedation. However, the feasibility and practicality of such a concept have to be evaluated in future studies.

There is still a lack of knowledge on the influence of intra- and peri-procedural measures on the outcome of the myoma ablation. Important peri-procedural aspects predicting treatment outcome after MR-guided HIFU are related to the immediate non-perfused volume ratio and other quantitative MRI parameters such as signal intensity ratio of fibroid to skeletal muscle in T2w MRI^24–26^.

**Figure 5:**
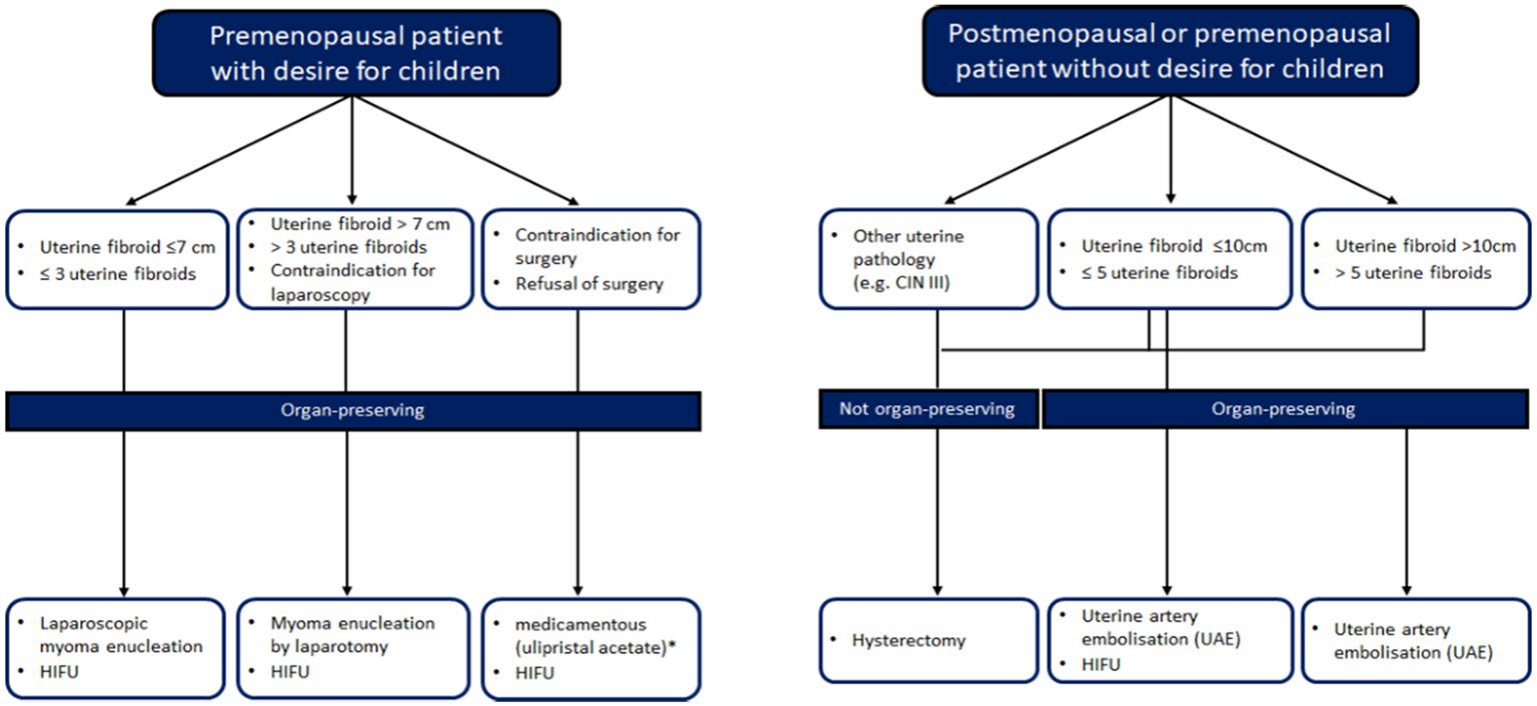
Treatment options for uterine fibroids.

From the gynecologist’s point of view, an international meta-analysis estimating the clinical benefit of HIFU treatment for women with uterine fibroids showed a comparable efficacy to myomectomy or hysterectomy and significant superiority to medical treatment with mifepristone^27^. In our study, HIFU was superior in terms of fever, transfusion, gastrointestinal tract, and anesthesia complications. Several studies showed that HIFU treatment of symptomatic uterine fibroids leads to comparable symptom improvement compared to laparoscopic procedures whereas HIFU shows fewer adverse events, shorter hospital stay, and faster recovery^28–30^. The findings of our study are in keeping with these results. The current German recommendations^1^ with regard to the different treatment options of uterine fibroids are summarized in Fig. 5. A recent survey of gynecologists reported that when surgery was suggested as first step, the preferred surgical treatments were myomectomy (71%) and hysterectomy (25%)^31^. This result is in line with our relatively small number of patients undergoing USgHIFU (n = 40 of a total number of 543 patients) at our institution. A clear-cut guideline of treatment recommendations as outlined above could contribute to a more differentiated medical approach in future.

In summary, US-guided HIFU treatment in patients with uterine fibroids is an effective therapeutic option which can be considered safe from an interdisciplinary standpoint. Analgesic treatment and body temperature management are critical points where an adequate cooperation of the intervention team is mandatory. More studies as well as innovative approaches are necessary to optimize peri-and postprocedural pain therapy.
